# Editors’ Choice—Review—The Future of Carbon-Based Neurochemical Sensing: A Critical Perspective

**DOI:** 10.1149/2754-2726/ad15a2

**Published:** 2023-12-27

**Authors:** Blaise J. Ostertag, Ashley E. Ross

**Affiliations:** 1 University of Cincinnati, Department of Chemistry, Cincinnati, Ohio 45221-0172, United States of America

**Keywords:** Neurotransmitter, fast scan cyclic voltammetry, carbon fiber microelectrode, carbon materials, dopamine

## Abstract

Carbon-based sensors have remained critical materials for electrochemical detection of neurochemicals, rooted in their inherent biocompatibility and broad potential window. Real-time monitoring using fast-scan cyclic voltammetry has resulted in the rise of minimally invasive carbon fiber microelectrodes as the material of choice for making measurements in tissue, but challenges with carbon fiber’s innate properties have limited its applicability to understudied neurochemicals. Here, we provide a critical review of the state of carbon-based real-time neurochemical detection and offer insight into ways we envision addressing these limitations in the future. This piece focuses on three main hinderances of traditional carbon fiber based materials: diminished temporal resolution due to geometric properties and adsorption/desorption properties of the material, poor selectivity/specificity to most neurochemicals, and the inability to tune amorphous carbon surfaces for specific interfacial interactions. Routes to addressing these challenges could lie in methods like computational modeling of single-molecule interfacial interactions, expansion to tunable carbon-based materials, and novel approaches to synthesizing these materials. We hope this critical piece does justice to describing the novel carbon-based materials that have preceded this work, and we hope this review provides useful solutions to innovate carbon-based material development in the future for individualized neurochemical structures.

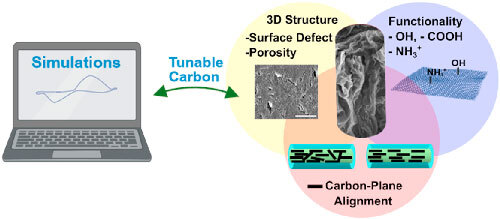

## Highlights

- Review limitations of carbon-fiber microelectrodes for neurochemical sensing.
- Future of tunable carbon-based sensor design.
- Academically-accessible synthesis approach needs.
- Need for molecular dynamic simulations of interfacial adsorption interactions.

Here, we provide a critical perspective on the future of carbon-based subsecond electrochemical detection of neurochemicals with fast-scan cyclic voltammetry (FSCV). We do so by acknowledging previous work that has revolutionized this field of study, what we perceive the main limitations that have been encountered to date are, and the possible ways these challenges can be addressed. The understanding of neurochemical communication has been widely acknowledged^1–3^ warranting a rise in carbon-based electrochemical sensing to provide a means to grasp the complexity of these processes.^4–7^ Neurochemicals play a vital role in sensory processing, motor control, behavior, etc, and their signaling functions to transmit chemical messages for these regulatory processes.^1,3^ By studying these processes we can provide critical insight into brain sensory processing and begin to interpret not only how our behaviors are controlled but also elucidate the causes of neurodegenerative diseases for improved treatment regimens. For several decades, real-time neurochemical detection has been dominated by electrochemical sensing.^1,6–9^ Carbon-based materials have taken over the field because of their biocompatibility, sensitivity, and ability to make rapid measurements for fast-signaling electroactive neurotransmitters;^10,11^ to date, carbon fiber microelectrode remains the gold standard for neurochemical monitoring in tissues due to the aforementioned properties.

The forefront of real-time electrochemical-based neurochemical sensing lies in the hands of FSCV at carbon fiber microelectrodes (CMFEs). FSCV enables subsecond monitoring of neurotransmitters and uses a minimally invasive carbon-based working electrode.^10,11^ Physically, carbon provides a geometry suitable for tissue implantation to measure biological processes at low, rapidly changing concentrations,^5,6,12^ but CFMEs are smooth with very little surface defects.^6^ Chemically, carbon’s broad working potential window enables extensive identification of a neurotransmitter’s unique redox interactions with high electron transfer rates.^13,14^ The innate oxide functionality on carbon fiber preconcentrates positively charged neurotransmitters, promoting enhanced adsorption kinetics.^5,15^ Despite this, CFMEs are amorphous with no way of isolating specific carbon functionalities. Some of these flaws in the carbon microstructure have led researchers to explore a variety of carbon nanostructures to enable manipulation of the neurochemical sensing platform. Various carbon nanostructures have been developed through carbon surface treatments to manipulate the surface geometry/chemical connectivity,^16–19^ modification additions of carbon nanostructures onto carbon fibers,^20–23^ or the synthesis of new carbon materials to focus on sought after sensor attributes.^24–27^ Nanomaterials possess innate properties (high surface area, electrical and mechanical properties, etc) capable of improving neurochemical measurement kinetics, sensitivity, and, at times, selectivity depending on the analytical technique of use.^28–30^ Carbon-based nanomaterials are unique with elaborate nanoscale geometries and chemical structures providing numerous useful applications for enhancing electrochemical detection of neurochemicals. All in all, there are many properties that carbon-based materials offer to advance neurochemical detection, but there are some weaknesses that need to be addressed to pave the way for a brighter future in neurochemical detection.

Electrochemically sensing neurochemicals has vastly improved over the last few decades as technology and our understanding of neurochemical processes has advanced. Although carbon fiber is the material of choice for FSCV, the previously mentioned flaws are of importance in this critical review. We intend to provide insight into the current state of the field and the future directions of carbon-based neurochemical sensing while paying tribute to the countless innovations that have propelled the field to its current state. Namely, this critical review will broadly discuss three key aspects identified as considerable challenges that must be combated to further advance detection capabilities: limited temporal resolution for measuring neurotransmitters due to adsorption interactions at the surface influencing the temporal limits of the measurement, deprived selectivity of understudied neurochemicals due to misunderstood interfacial interactions, and lacking controllability in surface chemistry tunability. We would like to acknowledge that many electrochemical techniques using carbon-based electrodes have been developed for neurochemical detection, and there are underlying advantages and disadvantages to all techniques. Acknowledging these challenges, the goal of our critical review is to focus solely on advancements in carbon-based materials for fast-scan cyclic voltammetry detection and how they are being used to combat these challenges.

## Current Challenges

**Figure 1.**
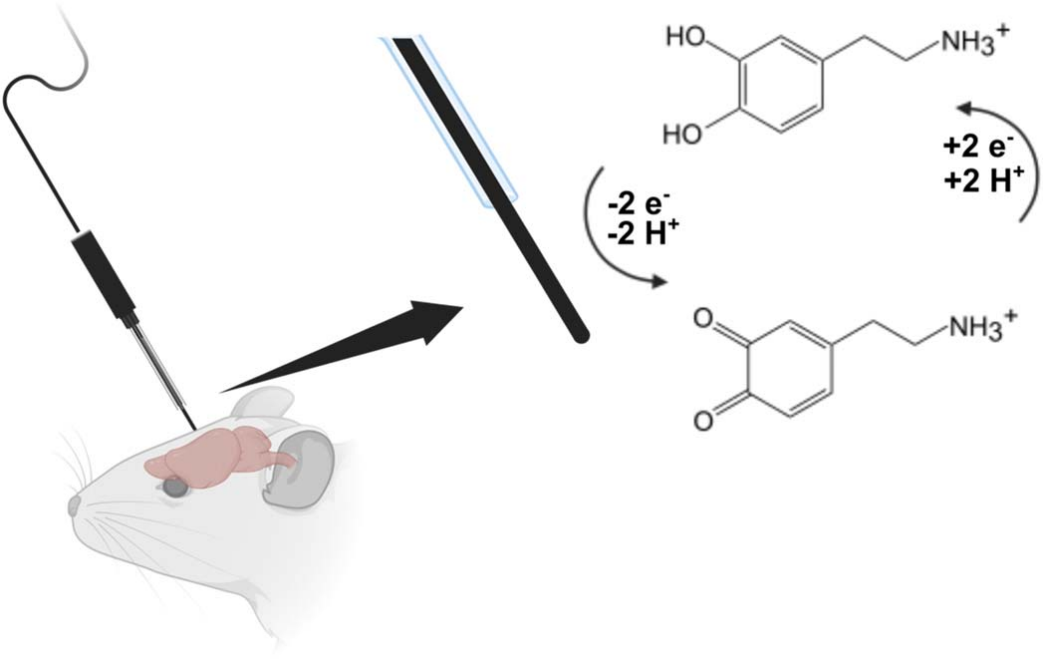
Schematic of in vivo detection of dopamine at a carbon-based microelectrode (created in Biorender.com).

Electrochemical detection at CFMEs have made a profound impact in neurochemical detection because of the ability to identify redox-active neurochemicals in low signaling concentrations while being minimally invasive to surrounding tissue. Given the nature of neurochemical interactions occurring in a biological environment, our understanding of their signaling has been vastly advanced through in vivo and ex vivo experiments, both of which require a biocompatible working electrode with micrometer dimensions. The traditional microelectrode of choice for ex vivo and in vivo measurements uses carbon fiber (CF). CFs’ dimensional and electronic properties have resulted in its surge to the forefront of real-time neurochemical detection with FSCV allowing for fast, sensitive neurochemical measurements, primarily of dopamine, in the brain (Fig. 1). Although CF is the gold standard for real-time neurochemical measurements, there are a handful of key aspects to carbon-based surfaces that must be improved for continued advancement of the field. We believe this call for change is rooted in the inherent chemical makeup of CFMEs, and, if these changes are addressed, the community will begin to make measurements of even greater temporal resolution on a substrate suitable for improved interfacial interactions tunable for various applications. Many recent advances have started to address these fundamental gaps and hindrances to the advancement of the neurochemical sensing field.^24–27^

## Unobserved Events Caused by Diminished Temporal Resolution

**Figure 2.**
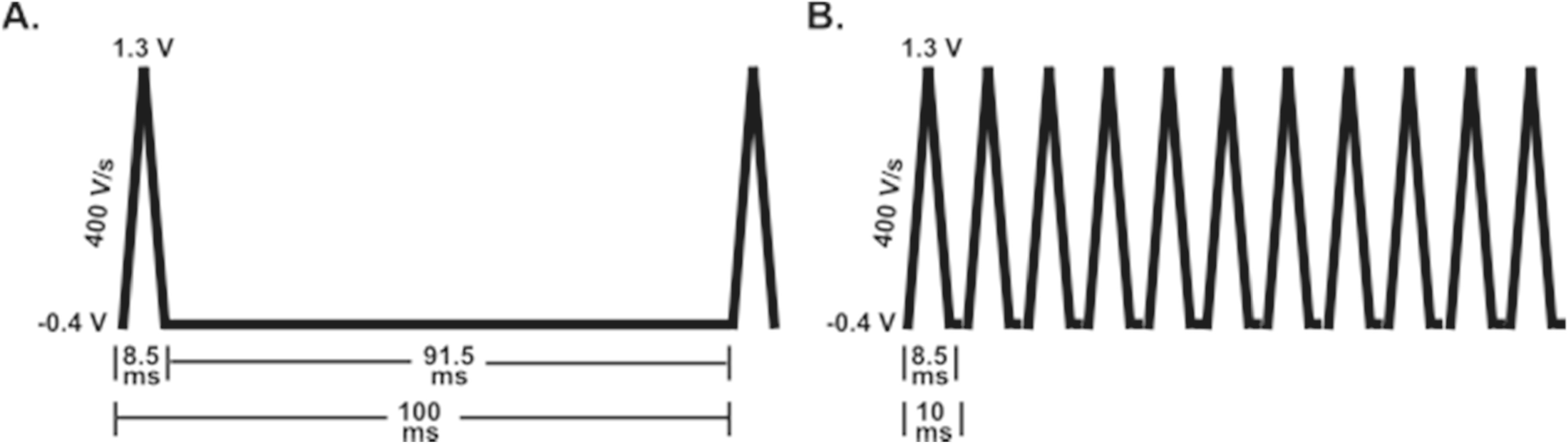
Schematic of the traditional fast-scan cyclic voltammetry waveform for dopamine scanning from −0.4 V to 1.3 V at a scan rate of 400 V s^−1^ and a frequency of (A) 10 Hz and (B) 100 Hz. Alteration of the application frequency has a major impact on measurement temporal resolution resulting in the idle time spent at the −0.4 V holding potential decreasing from 91.5 ms to 1.5 ms for application frequencies of 10 and 100 Hz respectively. These waveforms produce measurements of 100 ms (A) and 10 ms (B) temporal resolutions for dopamine detection.

Neurotransmitters signal on a millisecond timescale; therefore, specialized approaches are required to provide selective real-time sensing capabilities. Amperometry is a common electrochemical technique used to make temporally resolved measurements of neurotransmission, but, unless accompanied by a bio-recognition element to the electrode’s surface, amperometry can lack detection specificity in some cases. Compensation for ultra-fast, specific measurements has come in the form of analytical voltammetric methods as applied potential is varied at extremely fast scan rates while measuring current aligning with a redox-active molecule’s redox potentials for analyte identification.^10^ As selectivity is a key requirement for neurochemical identifiability, this critical review will focus on electrochemically detecting neurochemicals directly with voltammetry rather than indirectly with other techniques. As the prominent electrochemical technique used, FSCV at CFMEs is well known for its dynamic measurements of dopamine signaling with optimized voltammetric waveforms used to enhance dopamine-CF interactions. Although these experimental parameters are optimized, techniques like FSCV are dependent on adsorption interactions resulting in a temporal limit of 100 ms (Fig. 2A) to enable adequate dopamine adsorption at the surface to take place. The extended holding time required for dopamine adsorption results in optimized waveform application frequencies of 10 Hz preventing truly “real-time” monitoring of neurotransmission (Fig. 2A). Measurements made at application frequencies greater than 10 Hz are not possible without a significant loss in sensitivity because higher waveform application frequencies result in a diminished adsorption time, ultimately depleting dopamine’s surface concentration. Producing novel carbon materials capable of adequate adsorption at faster waveform application frequencies (like 100 Hz, leading to a 10 ms temporal resolution) would result in significantly improved temporal resolution. (Fig. 2B). To improve temporal resolution, many groups have explored the impact of carbon-based material geometry on neurochemical-electrode interactions to render the materials “frequency independent.”

### Novel work to this point

Over the years, an overarching debate has taken place as researchers attempt to define the term “real time” detection especially when it pertains to neurochemical sensing. Due to neurochemicals signaling on a millisecond timescale, we denote electrochemical methods to be operating in “real time” when detection is resolved on an equivalent time scale. Although optimized voltammetric methods operating at ultrafast scan rates still permit some signaling events to go unmeasured, researchers have used two broad methods to address and improve the temporal resolution of their measurement. These methods include replacing traditionally used carbon-based materials (i.e., CF) with new carbon-based materials to improve neurochemical-carbon interactions or introducing porous structures to carbon-based substrates to induce enhanced electrochemical reversibility and redox cycling.

**Figure 3.**
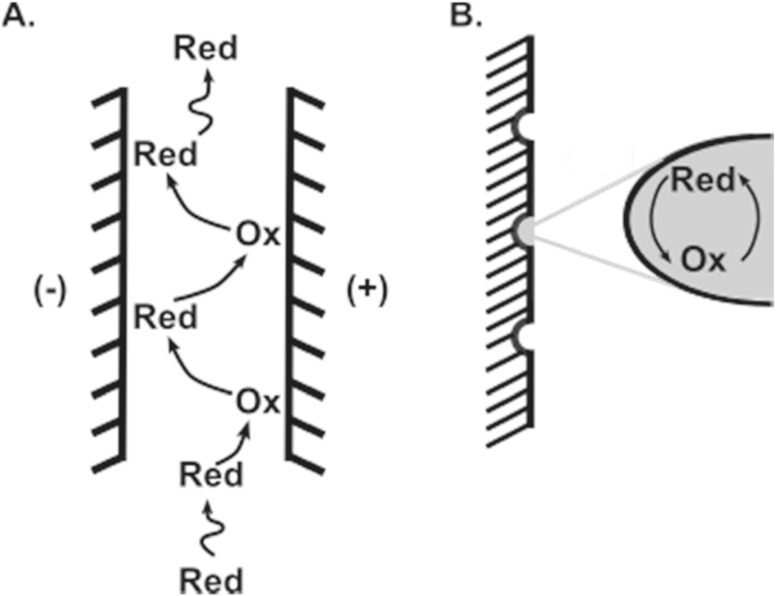
Schematic of individual electroactive small molecule surface interactions illustrating that as a small molecule (i.e., neurotransmitter) approaches a thin layer cell or porous electrode’s surface it is locally trapped allowing redox cycling and current amplification. The local trapping process permits recycling of the molecule producing local concentration increases at the electrode’s surface accompanied by current amplification. (A) Two biased electrodes of a thin layer cell promote a redox cycling mechanism for individual molecule current amplification. (B) Electrode’s with pore structures on the geometric scale of the molecule’s diffusion kinetics locally trap small molecules creating a thin layer cell—type environment during a fast voltammetric sweep creating a “biased potential” environment before molecules can desorb/diffuse away.

Thin layer cell electrochemistry has inspired novel carbon-based electrode fabrication in the neurochemical detection world as the concepts of local trapping, redox cycling, and low concentration current amplification are attractive to temporally resolved detection.^31^ Investigations have demonstrated that geometry plays a major role in creating thin layer cell type environments at carbon electrodes, especially at scan rates capable of real time neurochemical detection.^32^ CNTs exhibit properties to induce enhanced adsorption of redox-active molecules leading to improvements in electrochemical reversibility to the point of redox cycling for frequency independent, temporally resolved behavior.^24,33^ The concept of local trapping is produced by an electrode containing a porous geometric structure allowing an analyte of interest to be “trapped” within the structure causing it to be recycled within the thin layer cell/pore as the potential is biased or swept voltammetrically (Fig. 3). When the size of these porous structures is on the scale of diffusion kinetics of the neurochemical of interest, the local concentration is increased resulting in current amplification intriguing many researchers studying rapidly changing, low concentrations of neurotransmitters to introduce these structures to their sensing interface.

**Figure 4.**
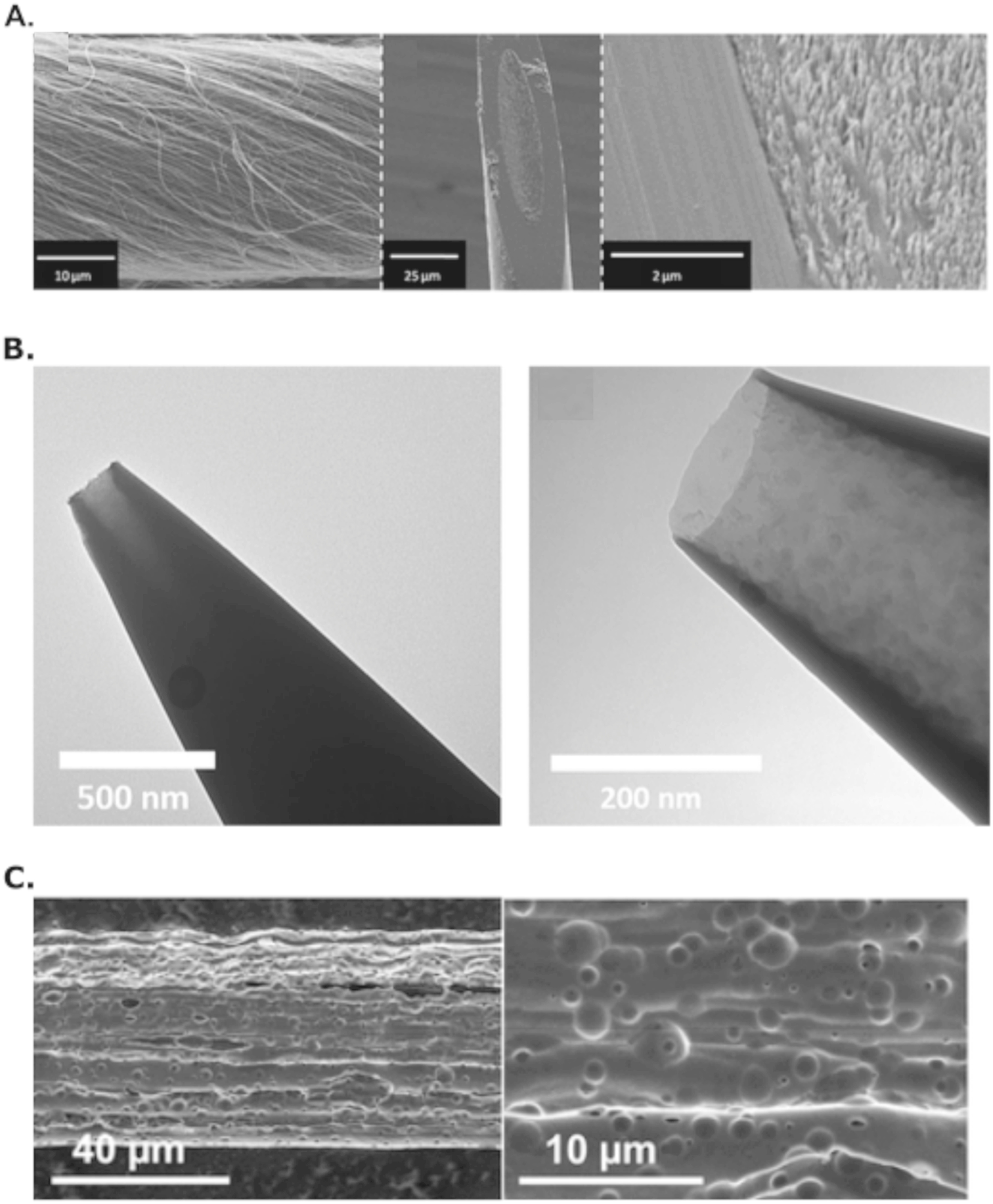
Example electron microscopy micrographs of various carbon materials found to promote voltammetric temporal improvements due to their geometry and/or chemical structure. (A) Scanning electron micrograph of carbon nanotube yarn. Reprinted with permission from Ref. 24. Copyright 2013 American Chemical Society. (B) Transmission electron micrograph of cavity carbon nanopipette electrode. Reprinted with permission from Ref. 26. Copyright 2019 American Chemical Society. (C) Scanning electron micrograph of copolymer-derived porous carbon fiber. Reprinted with permission from Ref. 25. Copyright 2023 American Chemical Society.

Although CF remains the gold standard for real time neurochemical detection today, various other carbon-based nanomaterials possess sought after properties that improve neurochemical detection through local trapping (Fig. 4). CFME surface modifications with carbon materials have gained popularity as porous geometries can be introduced through these material additions: carbon nanotubes,^34^ porous carbon nanofibers,^21^ carbon nanospikes,^20^ and several others. Carbon nanotubes (CNTs), when coupled to microelectrode frameworks, aid in mass transport enhancements with appreciable improvements in electrical conductivity and can even mitigate biofouling when implanted in tissue.^35^ Their highly sp^2^ hybridized composition consists of ends of abundant edge plane, sp^3^ sites suitable for electrochemical detection. These properties have resulted in CFME surface modifications with CNTs^34^ or the fabrication of CNT yarn electrodes (Fig. 4A) to replace CFMEs entirely.^24,33,36–38^ Neurochemical monitoring using CNTs improves various aspects of detection such as electron transfer rates, electrochemical reversibility, and temporal resolution. Through enhanced dopamine-CNT yarn interactions, more temporally resolved measurements can be made at both higher scan rates^24^ and application frequencies^27^ for faster data acquisition relying less on adsorption preventing unmonitored events. These changes in temporal resolution have been attributed to the inherent roughness of the electrode surface, introducing thin-layer cell like environments. More recently, our lab has explored graphene-based materials, specifically graphene oxide (GO). GO has a well-defined biplanar composition of high specific surface area offering immense electrical conductivity coupled with surface defects due to oxygen doping which reduces conductivity but improves edge-plane interactions of neurotransmitters.^39^ This utility has resulted in the fabrication of GO coatings and microfibers for neurochemical detection taking advantage of the highly admired basal plane step-edges and edge-plane adsorption driven processes^39–43^ to improve dopamine sensitivity under frequency independent detection conditions.^44^ Ultimately, these materials offer suitable improvements for temporally resolved real time neurochemical measurements in the absence of CFMEs.

The phenomenon of locally trapping neurochemicals has continued to lead to the development of other novel carbon structures through processes like chemical vapor deposition (CVD) and spinning dope alterations during carbon fiber synthesis.^25,26^ CVD has been used to deposit carbonaceous material within a nanocavity electrode (CNPEs) capable of locally trapping and amplifying the current of low concentrations of dopamine (Fig. 4B). This local trapping generates temporally resolved measurements similar to CNTs and GO fibers.^26,27,44,45^ Traditional CF is synthesized by a process called wetspinning, a process using an organic carbon-rich polymer that is carbonized and annealed to form an amorphous CF framework. Our lab has made recent developments to introduce secondary polymers that decompose during the heating process rendering macroporous geometries capable of improving measurement temporal resolution (not to the extent of CNTs or CNPEs) through porous geometries altering the surface chemical connectivity of these porous CFs (Fig. 4C).^25^ Although recent innovations have proven to improve on CF’s diminished temporal resolution, there are still limiting factors keeping researchers from discarding CF all together.

### Limitations & routes of addressing these challenges

CF has remained a staple to neurochemical sensing for decades as its biocompatibility and non-invasive nature provides viable tissue implantation. Unfortunately, many of the aforementioned nanomaterials dimensionally increase the electrode’s surface increasing its invasiveness; in the case of porous carbon microfibers or carbon nanotube yarns, electrode diameters begin to approach 20 *μ*m^25^ or even 30 *μ*m^33,36,44^ respectively as opposed to 7 *μ*m traditional CF^46^ reducing biocompatibility. Diminished tensile strength of these materials makes for structures too wide to pierce tissue or require disk electrode fabrication causing these electrodes to possibly break into tissue or have reduced depth of electrode exposure. Additionally, despite brilliant engineering, many of these synthesized nanomaterials require specialized equipment (i.e., reactors) or collaborations for their fabrication, rendering their synthesis challenging or impossible for researchers without access to this equipment. CF is even commonly synthesized on the industrial scale, so engineering is required in the fabrication process to construct lab friendly equipment for common lab practices. Ultimately, the temporal improvements brought by these innovative materials call for more implantation-friendly materials with more accessible fabrication processes.

Academic settings have resulted in researchers investigating simpler carbon fabrication processes to improve neurochemical detection. Several groups have developed simplified synthetic procedures for carbon-based fiber fabrication as possible CF replacements. The first procedure has abandoned CF entirely as graphene oxide (GO) composite coatings^40^ and microfiber frameworks^44^ have become an attractive material for neurochemical detection. GO can be purchased in dispersions allowing for simplified processes like mixing with positively charged polymers for charge balancing (i.e., PEDOT)^40^ or the hydrothermal method^44^ requiring only a mold for fiber formation and very low thermal treatments for drying. These fibers can be easily fabricated and promote temporally resolved neurochemical measurements. The second procedure, produced by the Venton lab, incorporates a coagulating polymer and carbon nanotubes for the formation of a wetspun fiber to replace CF as the microelectrode of choice.^38^ These polyethylenimine carbon nanotube fibers were fabricated on an in-house wetspinning setup and permit frequency independent measurements even while dopamine surface kinetics remain adsorption controlled. The third of these procedures required the construction of an in-house wetspinning apparatus where polyacrylonitrile (PAN) solutions were spun into plasticized, polymeric precursor fibers and treated in a low-priced tube furnace under inert conditions.^25^ This process enabled the usage of selected additives where a thermally decomposing polymer was strategically added to the PAN solution permitting pore generation upon thermal treatment. As an academic-accessible process, this simplified wetspinning procedure could open the door to new innovations in CF syntheses. These three synthetic approaches open the door for innovations we envision for academic friendly carbon-based material fabrication in the future.

Overall, novel carbon-based materials have been engineered to address neurotransmitter detection’s limited temporal resolution rooted in its common reliance on adsorption surface interactions influencing the temporal limits of FSCV measurements. We envision the future of carbon-based material syntheses incorporating the synthesis of full fiber frameworks, in contrast to CF modifications, to take advantage of these attractive surface geometries across the entirety of the sensing interface to further enhance the local trapping effect of adsorption-hindered neurotransmitters. Academic-setting-friendly fiber fabrication setups will enable individualized stretching and thermal methods to produce dimensional fibers on the scale of CF with improved elastic properties for implantation and alterations of their graphitization for varied molecular orientation or crystallinity. These advances will enable researchers to incorporate the study of mechanical properties, such as Young’s modulus,^18^ with surface geometry alterations on porous substrates to understand the impact these molecular orientations play in elaborate surface geometries. Future work involving polymeric solution additives could also be explored for higher dimensional porous geometries or even doping for enhanced fiber functionalization for increased oxygenation or introduction of new elements to CF’s framework. We have only begun to scratch the surface with these novel microfibers material advancements.

## Misunderstood Interfacial Interactions Leading to Poor Selectivity

**Figure 5.**
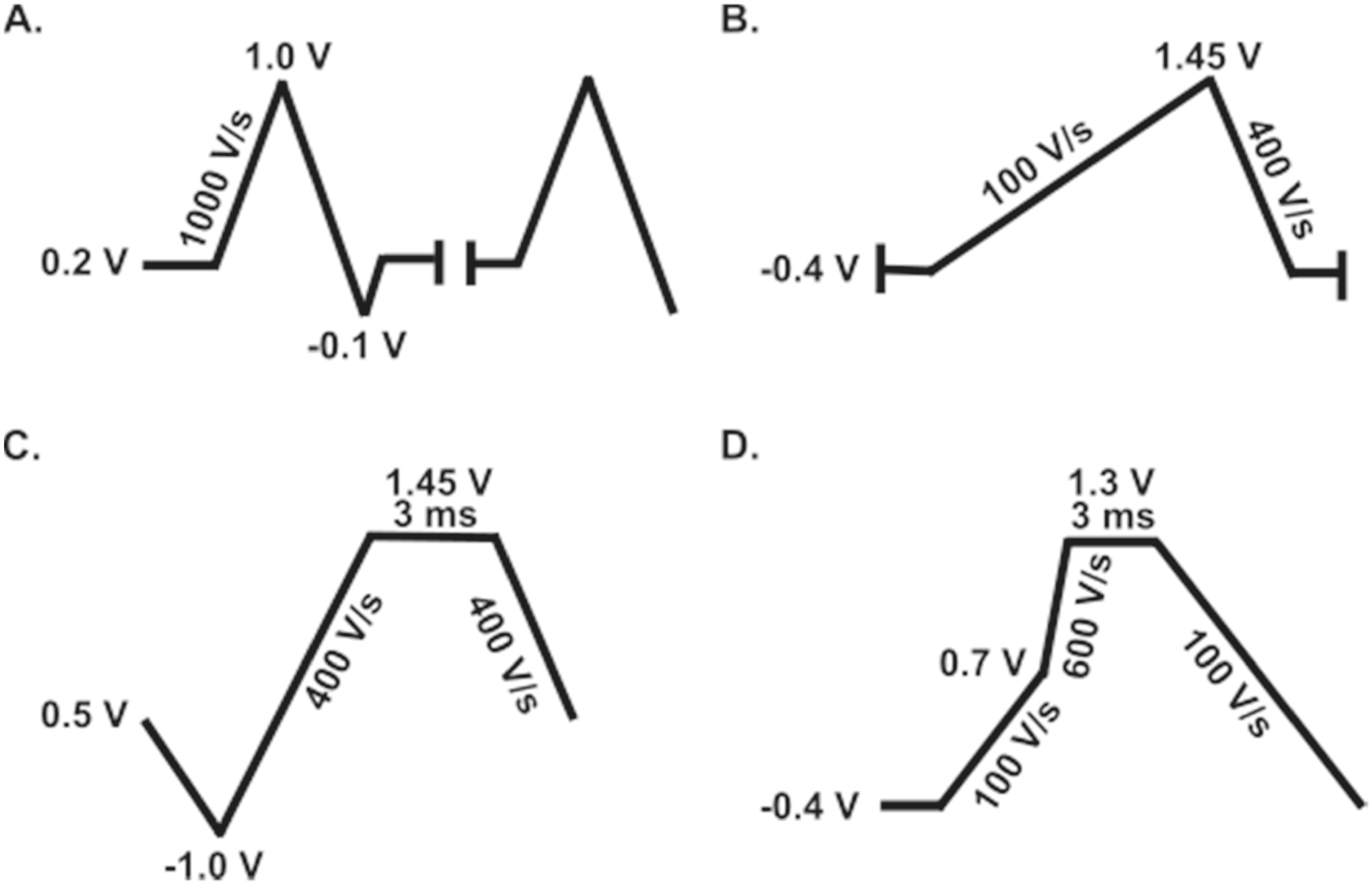
Waveform optimization has been shown to improve analyte selectivity of neurochemicals at carbon fiber microelectrodes. (A) Jackson waveform for serotonin detection Reproduced from Ref. 49. Copyright 2009 American Chemical Society. (B) Scalene waveform for guanosine and adenosine co-detection Reproduced from Ref. 53. Copyright 2019 American Chemical Society. (C) Extended sawhorse waveform for zinc detection Reproduced from Ref. 56. with permission from Springer Nature. (D) Multiple-scan-rate waveform for methionine-enkephalin detection. Reproduced from Ref. 57. Copyright 2022 American Chemical Society.

Traditional CF does not exhibit adequate selectivity for the detection of neurochemicals in complex matrices like the brain. Regularly, neurochemicals of differing redox potentials are identifiable by a scanning voltammetric waveform allowing for oxidation/reduction peak separation,^47,48^ but a key issue encountered with electrochemical detection is poor neurochemical specificity/selectivity.^49,50^ Techniques like FSCV encounter neurochemicals of equal redox potentials rendering them indistinguishable.^49^ Extensive research illustrates the biplanar nature of carbon-based materials:^39,51^ notably the sp^2^ hybridized basal plane offering predominately electronic conductivity and surface defects and the sp^3^ hybridized edge plane rich of oxide functional groups suitable for adsorption, electron density, and surface reactivity. Both planes play a major role in neurochemical surface interactions, but materials like CF have a purely heterogenous biplanar composition (amorphous). Although the challenge of specificity/selectivity in neurochemical detection is often combatted by waveform alterations in voltammetric studies (Fig. 5), innovative work has been done to explore and improve the interesting neurochemical-electrode interactions of molecules, especially for molecules beyond dopamine.

### Novel work to this point

If you were to ask anyone in the neurochemical detection community what the gold standard neurochemical to study is, chances are they would all give the same answer: dopamine. Dopamine has been vastly studied, and rightfully so, due to the major roles its signaling plays in regulating proper bodily function along with its ideal interactions at CFMEs. Dopamine’s importance is nothing to ignore, but the extent of research performed to study dopamine has partially been rooted in misunderstood interfacial interactions of other neurochemicals. In recent years, electrochemical detection of neurochemicals has slowly branched out to other classes. Unfortunately, CF’s amorphous composition has prevented the concept of surface chemistry-selective electrodes leaving selectivity in the hands of voltammetric waveform modifications or biorecognition element additions.

Voltammetric waveform modifications cancompensate for CF’s amorphous surface chemistry and difficulty in manipulation for enhance selectivity to neurochemicals beyond dopamine (Fig. 5). Serotonin, a neurochemical with a known oxidation potential like that of dopamine, lacks selectivity when in the presence of the well-studied catecholamine, and serotonin is also known to foul the electrode’s surface hindering its detection. The Jackson waveform (Fig. 5A) was developed to reduce serotonin fouling, and, in doing so, improved serotonin selectivity as the applied potentials were altered from an optimal dopamine waveform.^52^ Here, a holding potential of 0.2 V negates dopamine’s positively charged adsorption interactions at the CF’s surface at negative holding potentials, creating selective serotonin detection while the addition of a cation exchange polymer on the electrode prevents adsorption of other competing neurochemicals. One class of neurochemicals, purines, has been shown exceptional interest in recent years,^15,19,23,47,48,53–55^ particularly adenosine and guanosine, for their neuroprotective roles during brain injury. Several voltammetric waveforms have been optimized for adenosine, guanosine, and even co-detection (Fig. 5B) of both neuroprotective neurochemicals.^48,54–56^ Additionally, extensive work was performed on histamine, an important neuromodulator, to vastly investigate its oxidation mechanism using amperometry and traditional cyclic voltammetry for improved FSCV detection and to also improve our understanding of its electropolymerization fouling mechanism.^57^ Numerous other studies have been performed to improve selectivity to various other neurochemicals, even metal neurotransmitters like zinc (Fig. 5C).^58,59^ Waveform modifications have also been used for resolved, dynamic detection of opioid peptides, namely methionine-enkephalin, which is implicated in stress, pain, and reward (Fig. 5D).^60^ Although effective, the majority of these solutions have one thing in common: all involve manipulation of the voltammetric waveform and overlook the need to understand these neurochemical’s interfacial interactions at carbon surfaces.

**Figure 6.**
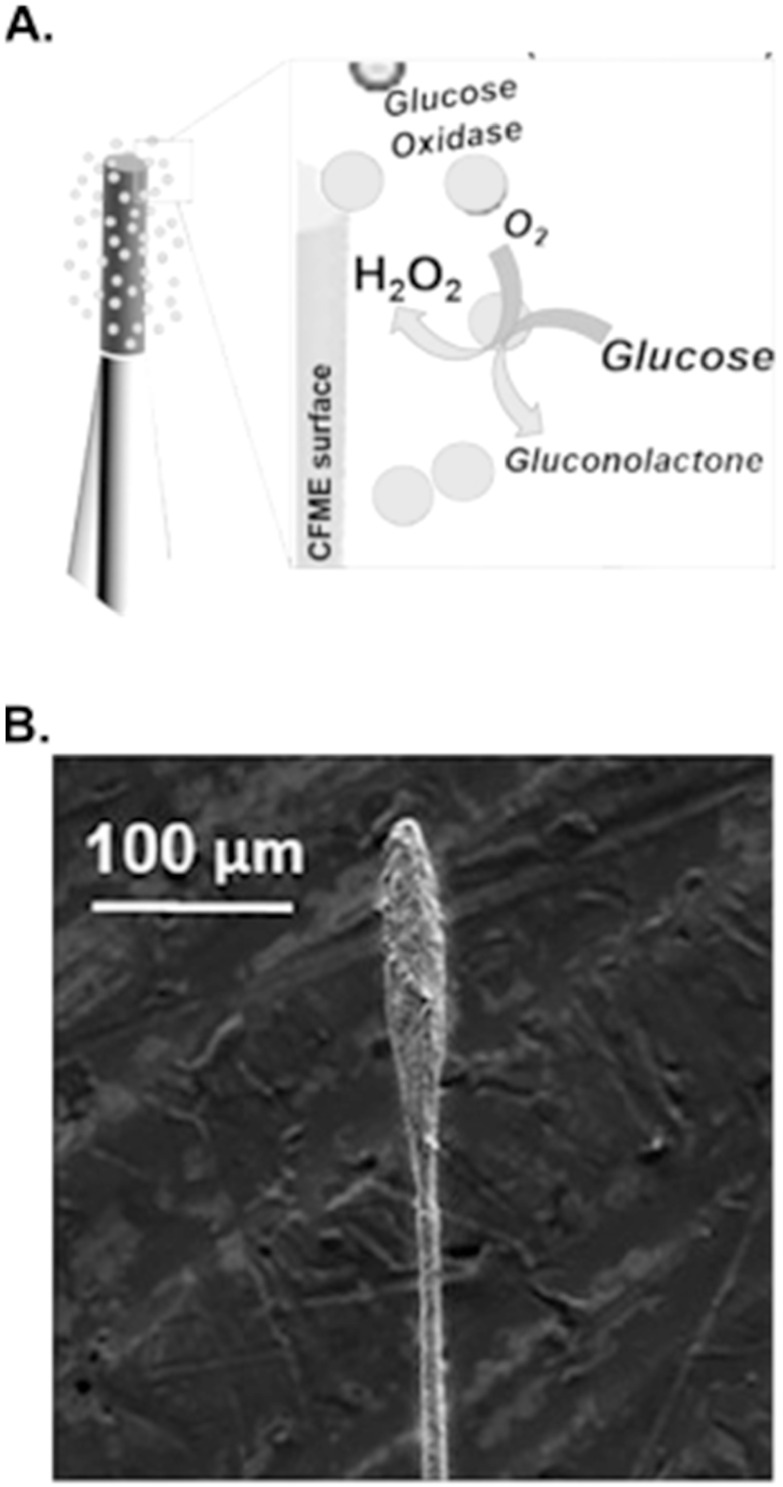
Biorecognition modified electrodes show highly selective electrochemical detection of non-electroactive neurochemicals enhanced by the introduction of selective enzymes to the electrode’s surface and detection of electroactive hydrogen peroxide. (A) Glucose oxidase enzyme modified CFME. Reproduced with permission from Ref. 58. Copyright 2016 American Chemical Society. (B) Lactate oxidase modified CFME. Reproduced with permission from Ref. 59. Copyright 2018 American Chemical Society.

Routes to increase CFME selectivity without a predominant focus on voltammetric waveform alterations have turned to biorecognition elements in the form of enzyme-modified CFs. Enzymes can be highly specific for neurochemical detection and combative for interferents. These improvements provide advancements in detection selectivity and surface interactions of many neurochemicals hindered by the non-selective nature of unmodified, amorphous CF (Fig. 6). FSCV is an electrochemical technique known for its capability to only measure electroactive analytes, one of the largest limitations to the technique. By modifying the CFME with glucose oxidase enzyme (Fig. 6A), the Sombers group has measured non-electroactive glucose by enzymatically generating electroactive hydrogen peroxide which is oxidized and correlated to the present concentration of glucose at the modified electrode surface.^61^ This innovative enzyme-modified electrode expands the capability of voltammetric measurements using CFMEs enhancing co-detection of electroactive (dopamine) and non-electroactive species (glucose). The Sombers group also engineered a lactate oxidase-modified CFME to selectively measure lactate using FSCV expanding our knowledge of lactate’s brain dynamics (Fig. 6B).^62^ The use of this modified CFME once again generates hydrogen peroxide enzymatically enabling electroactive analyte detection when lactate and molecular oxygen is present at the electrodes surface. Some researchers have even used non-carbon materials^63–69^ to expand on the concept of enzymatically detecting non-electroactive species with FSCV. The Venton lab has fabricated a glutamate-selective biosensor on a platinum-based electrode.^64–69^ Future work could expand toward improving glutamate sensing with FSCV on carbon substrates. These novel micro-biosensors extend the capability of electrochemically detecting non-electroactive neurochemicals in real-time. Despite these important advancements, much more is needed to improve direct detection of a variety of electroactive neurochemicals including purines, indolamines, etc.

### Limitations & routes of addressing these challenges

Understudied neurochemicals suffer from misunderstood interfacial interactions, and the methods to address measurement selectivity, while effective, create unexpected limitations correlated to their modifications. Waveform modifications are optimized for selective detection of a neurochemical of interest, but these waveforms only alter the potential sweep applied to CFME while not addressing electrode composition or investigation into the neurochemical’s interfacial interaction. While these measurements are still made on amorphous CF, numerous neurochemicals rely on adsorption for their detection, and these surfaces are prone to non-specific adsorption and biofouling which block interfacial interaction sights decreasing the possibility of neurochemical detection at these potential ranges. The redox mechanism of some neurochemicals has proven detrimental for similar reasons as they electropolymerize through radical formation and foul at electrode’s interface.^42,52,57^ Waveform modifications ultimately do not address interferents/biproducts present in tissue matrices and can even hinder measurement dynamics,^56,59^ so we must begin to address the material making these measurements: CF. A novel route to accomplishing this includes the use of biorecognition elements, notably enzymes, for improved surface interactions with strong analyte specificity and selectivity.^61–63^ We envision biorecognition elements playing a key role in the future of dynamic neurochemical detection to distinguish coalesced redox peaks and further enhanced analyte specificity. We also hypothesize that as novel carbon materials of controllable surface chemistry are synthesized, the field will begin to fabricate biosensors like the glutamate oxidase enzyme sensor with carbon-based electrodes rather than metals like platinum. Soon we could even begin to see the inclusion of aptamers on microelectrode frameworks for selective neurochemical binding to continue to combat non-specific adsorption/biofouling. Ultimately, we believe the use of biorecognition elements could be the key to overcoming misunderstood interfacial interactions of neurochemicals to further improve analyte selectivity.

Innovative enzyme-modified electrodes enhance surface interaction and analyte selectivity resulting in individualized, compatible neurochemical detection. Because of this, there is a call for more biorecognition elements to improve neurochemical distinguishability. Electrochemical aptamer-based (E-AB) sensors are a biosensor used for in vivo measurements capable of real-time binding to a specific target molecule and rely on reporter binding rather than target reactivity.^70^ E-AB sensors have proven effective employing a redox reporter for continuous monitoring of therapeutic drugs,^71^ proteins,^72^ and others in biological fluids. Commonly, these sensors are fabricated on a gold substrate showing recent innovations on gold microneedles for in vivo implantation,^71^ but these have even shown viability on carbon substrates like glassy carbon^73^ paving the way for possible applications in FSCV. Their high affinity and specificity for their target molecules could prove useful on carbon microelectrodes allowing selective neurochemical detection. Although their selective nature is very attractive, using E-AB sensors universally would require a great deal of optimization for minimally invasive tissue studies. First and foremost, these sensors would need to be designed on a lower diameter scale for implantation as many micro—E-AB sensors have diameters orders of magnitude larger than traditional CF.^71,74^ Second, E-AB sensors are constructed by self-assembled monolayers. Depending on the thickness of the blocking monolayer and the conformation of the redox reporter for specific target molecules, certain E-AB sensors can exhibit hindered electron transfer,^70^ non-specific binding of the redox reporter, and slowed temporal resolution. Developing a means to scale E-AB sensors down to the scale close to that of traditional CF and using methods to prohibit non-specific binding, E-AB sensors could become extremely useful biorecognition elements for neurochemical detection in the future.

## Lacking Controllability in Surface Chemistry Tunability

Voltammetric techniques at scan rates capable of detecting neurochemicals in real time commonly rely on adsorption for effective interfacial interactions for analytes of interest. Adsorption processes themselves are more poorly understood than common diffusion processes, and this is largely due to little investigation into the fundamental mechanism of adsorption for many neurochemicals of interest. Understandably so, researchers have found amorphous CF’s surface lacking the ability to isolate specific surface chemical connectivity, but the introduction of tunable carbon surfaces could be the solution to surface sensitive neurochemical detection. The possibility of readily tuning a carbon substrate’s surface through geometry or chemical connectivity would allow for individualized surface interactions for the vastly different molecular structures of many of these neurochemicals. Fine tuning surfaces could come in the form of altering carbon hybridization for differing surface electroactivity, elemental doping for controlled surface functionality, or replaced amorphous CF in its entirety for more suitable substrates capable of this fine-tuning ability. There are numerous neurochemical classes comprised of molecules with similar chemical structures. What is odd is that many of the neurochemicals within these classes have slightly deviated chemical structures but have vastly different interfacial interactions generating a wide array of sensitivities to structurally similar molecules. Not only this, but some neurochemicals like purines also exhibit a multi-step oxidation scheme where various oxidation products present differing adsorption strengths requiring further investigations into suitable carbon materials for improved adsorption interactions to overcome desorption kinetics. Acknowledging that tunable carbon surfaces for neurochemical detection will result in individualized structures for the slightest differences in molecular structure, we would like to present a breakdown of many of these neurochemical classes and the novel work that has been done for each.

### Novel work to this point

**Figure 7.**
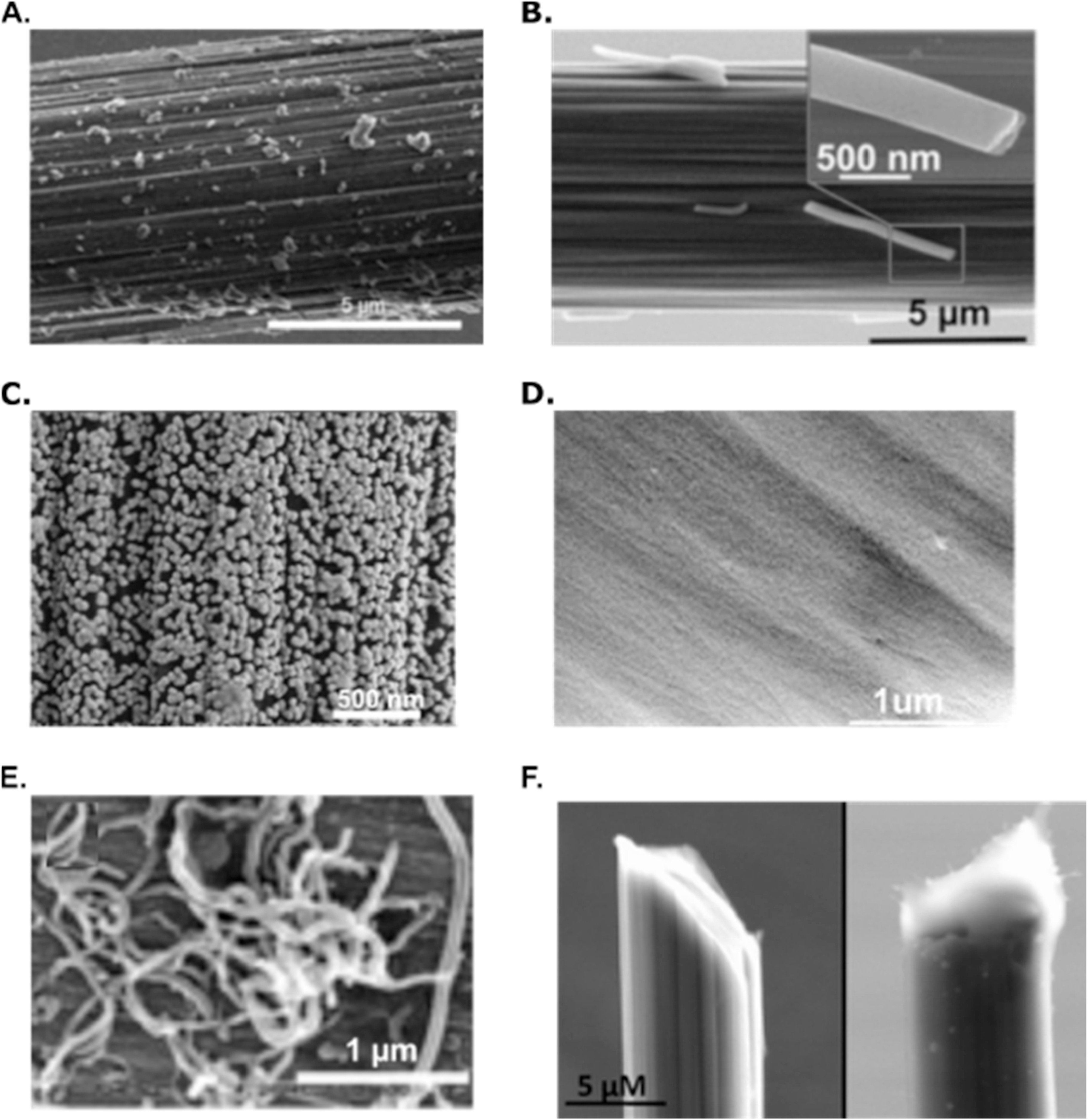
Carbon-based microelectrode nanoscale surface modifications have been shown to improve sensitivity, electrocatalysis, and reduce fouling of neurochemicals tuning the electrode’s surface for enhanced surface chemistry-driven interactions. (A) Graphene oxide-modified carbon fiber. Reproduced from Ref. 40 with permission from the Royal Society of Chemistry. (B) Porous carbon nanofiber-modified carbon fiber. Reproduced from Ref. 21. Copyright 2022 American Chemical Society. (C) Gold nanoparticle modified carbon fiber microelectrode. Reproduced from Ref. 22. Copyright 2022 American Chemical Society under license CC-BY-NC-ND-4.0 with no additional changes. (D) Nitrogen plasma-treated carbon fiber microelectrode. Reproduced from Ref. 15 with permission from the Royal Society of Chemistry. (E) Carbon nanotube-modified carbon fiber. Reproduced from Ref. 31 with permission from the Royal Society of Chemistry. (F) Nafion-coated carbon fiber. Reproduced from Ref. 49. Copyright 2009 American Chemical Society.

A repetitive topic of discussion throughout this manuscript is the low defect, smooth, amorphous nature of carbon fiber. Our understanding of neurochemical signaling wouldn’t be where it is today without the use of CF in analytical voltammetric methods, but amorphous materials hinder surface enhancements creating poor controllability in surface modifications and inability to easily tune the surface. CF is also prone to biofouling, a major concern when attempting to actively measure neurochemical signals in a tissue matrix. This may be attributed to the chemically variable surface offering abundant sites for the attraction of compounds like hydrophobic proteins. Compensating for amorphous characteristics, surface modifications have been prominent for enhancing CF surfaces (Fig. 7); methods have been developed to compensate for the needs of many different neurochemicals for increased sensitivity through what little controllability in surface tunability CF has at its disposal. A previous topic of discussion for improving sensitivity came in the form of voltammetric waveform modifications, and, for this reason, here we intend to solely focus on sensitivity improvements created by carbon surface modifications. This breakdown will make evident the need for improved modification robustness for carbon-based materials in the form of fully tunable fibers for individual neurotransmitter adsorption interactions.

#### Catecholamines

Catecholamines, released by the brain and adrenal glands, are hormones acting in response to physical and emotional stress.^75^ As a neurochemical class consisting of dopamine, catecholamines are the most well-studied neurochemical class when it comes to FSCV measurements. The catecholamine class is comprised of neurochemicals like dopamine, norepinephrine, and epinephrine, but a quick review of innovative FSCV advancements when it comes to these neurochemicals would show a predominant focus on dopamine despite its nearly identical molecular structure. The addition of a single hydroxyl group for norepinephrine generates an identical redox profile with nearly a 2-fold decrease in sensitivity as opposed to dopamine only explained by differing adsorption interactions dictated by slight variations in molecular structure.

A well-established proposal in the FSCV community is that catecholamines effectively adsorb to oxide functionality generating a wide understanding of dopamine’s interaction at oxide-rich carbon surfaces.^6,11,12,51,76^ Due to surface oxygen’s importance, various methods have been used to enhance CF’s oxide functionality to further improve catecholamine adsorption for improved FSCV detection.^15,76,77^ Electrochemical conditioning CF electrodes with extended waveforms have confirmed increases in hydroxyl and carbonyl functionality to the surface contributing increased dopamine detection sensitivity and electron-transfer kinetics.^76^ Similarly, using oxide-rich agents such as KOH for electrode pretreatment provides surface activation and increasing carbon surface regeneration rates to maintain sensitivity while decreasing biofouling resulting from oxide functionality increases on renewed CF surfaces.^77^ Additionally, methods like plasma treatment increase surface oxide functionality along with surface defects/roughness for enhanced dopamine detection.^15^ Surface oxygen functionality plays a vital role in catecholamine adsorption, but studies have also shown the importance of carbon surface roughness/defects to these interactions too.

**Figure 8.**
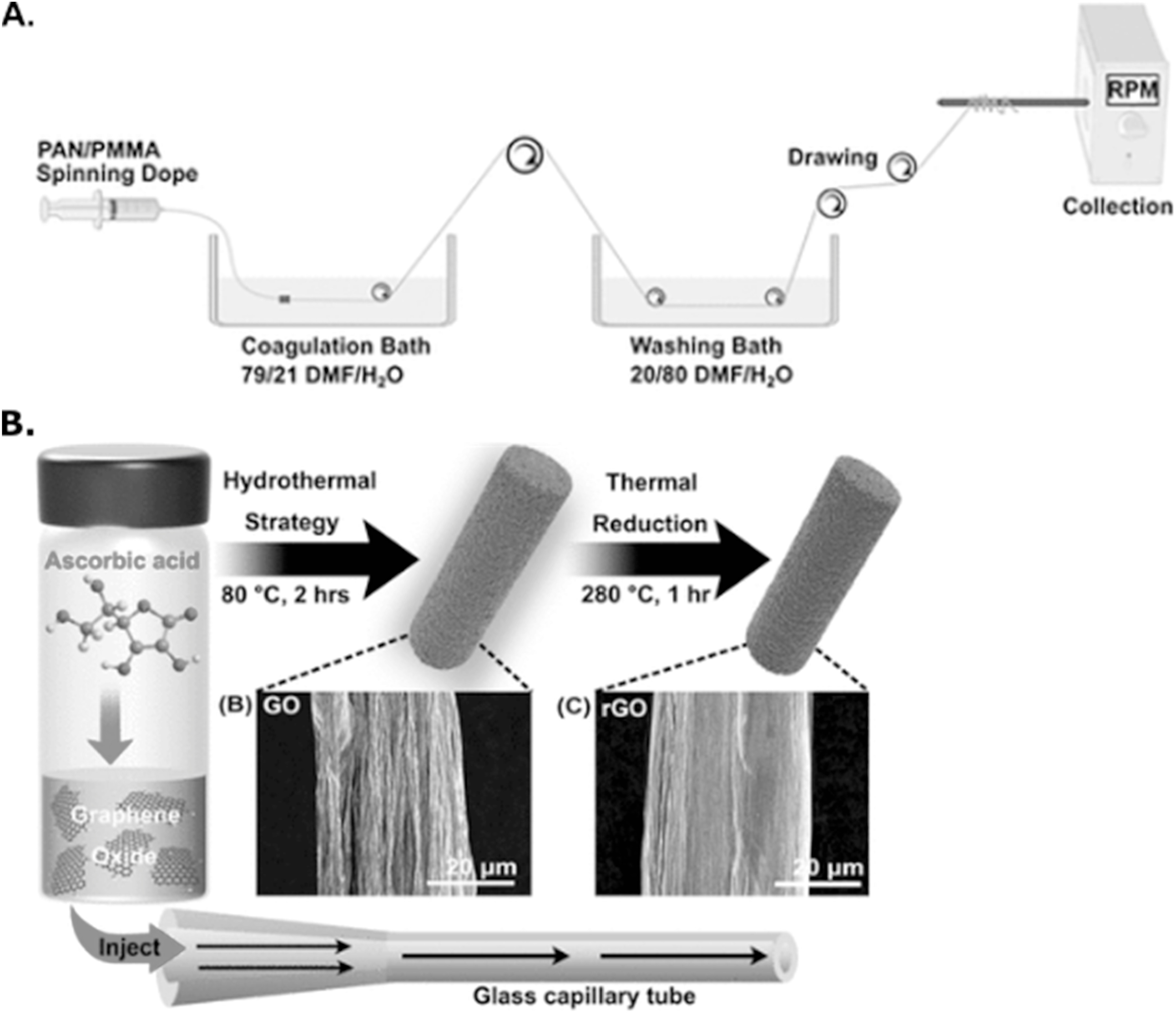
Highly tunable synthetic methods can form carbon-based material structures with controllable, attractive surface geometry/chemical connectivity. (A) Academic-scale copolymer wetspinning method for porous carbon fiber synthesis. Reproduced from Ref. 25. Copyright 2023 American Chemical Society. (B) Hydrothermal method for graphene oxide/reduce graphene oxide fiber synthesis. Reproduced from Ref. 41. Copyright 2022 American Chemical Society.

Tuning CF surfaces for improved dopamine detection has relied predominately on surface modification techniques such as dip coating,^19,52^ drop casting,^78,79^ electrodeposition,^15,21,23,43,80^ CVD,^20,24,26,27,33,81,82^ and plasma treatment.^15,16^ Although effective, many of these modifications tend to lack robustness and efficient reproducibility due to poor electrostatic and pi-pi interactions of carbon materials onto a carbon substrate. Carbon nanomaterials like graphene oxide (Fig. 8A),^43^ porous carbon nanofibers (Fig. 8B), CNTs,^24,34,35^ carbon nanospikes,^20^ and cavity carbon nanopipettes (Fig. 4B)^26^ have all shown enhanced surface roughness and surface defects; these enhancements improve detection sensitivity, kinetics, and can even produce a cyclization-induced secondary peak for some catecholamines^26^ through local trapping when modified onto the CF surface. These carbon nanomaterials have played a key role in advancing our understanding into the attractiveness of optimizing the geometric structures of carbon-based electrodes due to the influence of not only surface chemistry but surface geometry. While surface oxidation and geometry are vastly important for neurochemical surface interactions, we believe the importance of carbon hybridization for electrochemical applicability calls for a tunable substrate capable of manipulating the biplanar nature of carbon more robustly.^39^

#### Purines

Purines are heterocyclic molecules consisting of a fused pyrimidine and imidazole ring, and they are released throughout the nervous system aiding in neuromodulation, neuroprotection, and neuroinflammation. A large and diverse neurochemical class, we have shown that purines exhibit vastly different adsorption interfacial interactions depending on the various functional groups attached to this bi-cyclic structure.^53^ Although our lab has conducted an in depth study of how specific purine functional groups interact at CF surfaces,^53^ translating this understanding to new materials remains challenging due to the amorphous nature of the CF material. Our previous study showed not only what functional groups are favorable for CF substrates but also showed what electrode characteristics are required for purine-specific electrode development.^53^ By exploring a survey of purine structures, our group was able to show stronger surface interactions with greater adsorption and high surface coverage of amine-containing purines whereas triphosphate- and ribose-containing purines exhibit lower sensitivity and slowed interactions at amorphous CF. Despite CF’s oxide-rich surface, surface modifications have proven beneficial for improving adsorption interactions of those non-amine containing purines with otherwise unfavorable functionality. One recent innovation to improve sensitivity to these poorly interacting molecules is to modify the CF surface with metal nanoparticles for electrocatalytic improvements through neurochemical-metal interactions. Electrodepositing gold and platinum nanoparticles onto CFMEs have shown improved electron transfer rates to adenine-based purines allowing for improved detection sensitivity (Fig. 7C).^22,23^ Although these metal nanoparticles play major electrocatalytic roles in neurochemical detection, metal nanoparticles are known to be non-biocompatible and toxic if left in the body,^83^ so they pose a possible health hazard when implanted in living tissue. Additionally, we have shown that altering CF’s surface functionalization through nitrogen doping (Fig. 7D) can improve electrocatalytic conversion of purine oxidation products and improve adsorption of triphosphate/ribose-containing purines.^15,17^ Despite these advancements in our understanding of purine-carbon interactions, these modification approaches suffer from robustness issues mostly attributed to the amorphous nature of the CF substrate. A push to move to materials which are much more readily tunable with these changes in surface chemistry are needed.

#### Indolamines

Indolamines are neurochemicals consisting of an amine-substituted indole compound and two of the most common are serotonin (roles in sleep and mood) and melatonin (circadian rhythm and inflammation functions). Many studies on improving sensitivity to indolamines have used voltammetric waveform modifications, but there have been a few surface modifications that have proven helpful. A common challenge to studying indolamines, though, is that indolamines like serotonin have a similar oxidation potential to catecholamines and indolamines are prone to foul the electrode’s surface depleting voltammetric responses rendering electrodes unusable after a short period of time. The inability to isolate and expose specific planes of amorphous carbon creates issues in understanding the mechanism of redox generated, electropolymerized side product adsorption to the electrode surface.^18,84^ To overcome this, new frameworks have been employed in the form of carbon nanotube^35,84^ and graphene fiber^44^ microelectrodes utilizing their well-defined, biplanar chemical connectivity and electrocatalytic properties for fouling resistant surfaces. These concepts have also found utility with amorphous CF through surface modifications with CNTs for a similar anti-fouling effect (Fig. 7E).^34,85^ In terms of surface treatments, for example, the ion exchange polymer Nafion has been used to uniformly coat the electrode’s surface aiding adsorption and sensitivity,^52^ but charged polymers repel biomolecules of like charges and slow response times (Fig. 7F). Overall, further understanding of indolamine signaling in the future will rely on 1) mitigating fouling to combat electropolymerized side products from the electrode’s surface and 2) generating surface sensitive, tunable carbon substrates for improved measurement sensitivity.

#### Neuropeptides

FSCV has been employed for detecting large molecules such as neuropeptides. Neuropeptides are small peptides consisting of a short chain of amino acids, packaged in dense core vesicles, and released from neurons.^86^ Unfortunately, many of the amino acid residues which make up neuropeptides are not electroactive, which requires innovative sensing surfaces for measuring their release and specific enhancement for detection of the electroactive amino acids (namely tyrosine and tryptophan). This has resulted in the FSCV community to begin exploring real-time detection of several of these neuropeptides, one of which is pulsatile gonadotropin-releasing hormone (GnRH) which regulates fertility.^87^ The Venton lab has pioneered optimized GnRH detection in vitro followed by stable, sensitive measurement ex vivo.^87^ Their novel study has expanded our understanding into how GnRH is released and provides insight into how GnRH is functioning as a neuromodulator.^87^ The Somber’s lab has pioneered detection of enkephalin neuropeptides through the oxidation of ionizable tyrosine and methionine at CFMEs, resulting in the detection of methionine^60^ and leucine-enkephalin.^88^ The presence of tyrosine fouls the electrode which is combatted by modified waveforms (Fig. 5D).^60^ Our lab has performed an extensive study on all the electroactive amino acids to understand mechanisms of how their chemical structure interacts are at carbon fiber.^89^ We show the presence of sulfur-containing functional groups hinder amino acid—carbon fiber interactions, and we provide possible waveform design insight for amino acid-containing peptides.^89^ Overall, FSCV at CFMEs has provided a method for dynamic detection of these complex electroactive neuropeptides, but unattractive carbon surface interactions exist with challenges in selectivity suggesting that the use of bio-recognition elements could be warranted in the future.

#### Reactive oxygen species

Reactive oxygen species like hydrogen peroxide (H_2_O_2_) have been shown to act as a neuromodulator regulating rapid dopamine release in the brain.^90,91^ When in excess, H_2_O_2_ causes oxidative stress tying it to numerous neurological diseases.^90,91^ Discussed previously, several non-electroactive molecules are able to be detected electrochemically with the help of substrate-specific enzyme modified electrodes using H_2_O_2_ as a reporter molecule for indirect detection.^61,62^ Pioneering FSCV detection of H_2_O_2_ at CFMEs,^91^ the Sombers lab has provided us with a novel approach for sensitive detection in the dorsal striatum^92^ and have shown mechanistic studies into the production of hydroxyl radical intermediates and how this modulates dopamine dynamics.^93^ Overall, further investigations into H_2_O_2_’s surface interactions could give insight into favorable carbon structures/functionality to further improve detection sensitivity to vastly expand indirect non-electroactive molecule monitoring.

#### Metal neurotransmitters

Trace metals have been examined environmentally, but recent studies have proven their unique ability to act as neurotransmitters in the brain. Although knowledge of their roles as neurotransmitters is relatively new, many of these trace metals are important in neurological diseases creating excitotoxicity in high concentrations with ties to Alzheimer’s^94^ and Parkinson’s disease.^95^ Metal neurotransmitter detection with FSCV has found utility most notably for two key neurotransmitter, copper and zinc, to measure their rapid extracellular release. CFMEs accompanied by surface pretreatment have been employed to measure free copper providing insight into the adsorption interactions of the metal neurotransmitter at carbon surfaces.^58^ By electrochemically pretreating with a “copper sensitive” triangular waveform and chemically pretreating with sulfuric and nitric acid, this work made evident the adsorption surface mechanism pioneering FSCV detection of metal neurotransmitter analysis. Zinc, known to be co-packaged and co-released with glutamate, has drawn recent interest from our group due to its importance in co-signaling with glutamate. Zinc is known to plate carbon surfaces, so, to maintain sensitivity to rapid zinc detection, we developed a waveform to strip plated zinc after reduction with FSCV. Despite our work, we demonstrate the poor sensitivity of CFMEs to Zn detection, which directly supports the need to develop specific carbon-based materials which improve detection while minimizing irreversible adsorption. Overall, we have only scratched the surface of understanding the mechanisms of these metal neurotransmitters, and novel carbon materials could present a route to further our understanding their roles as neurotransmitters.

#### Imidazole

An imidazole is a structural class consisting of a five membered heterocyclic ring made up of three carbons and two nitrogens. The most common neurotransmitter within this imidazole structural class is histamine, known as an inflammatory agent and sleep cycle regulator. When investigating histamine electrochemically, it is drastically hindered by imidazole ring electrochemical oxidation which produces a radical, dimerizes, and then electropolymerizes rapidly on the CF electrode’s surface.^57^ The Venton group coupled various techniques to deduce the mechanism of histamine oxidation and electropolymerization. X-ray photoelectron spectroscopy provided elemental and electronic state information used to confirm polymerization of the oxidation products. Electrochemically, FSCV detection witnesses immense fouling of the CF’s surface which can be mitigated by Nafion coating. The extensive analysis presented in this work illustrates a blueprint for mechanistic analysis of the redox and polymerized biproducts of neurotransmitters. Coupled to these mechanistic studies, we would like to reiterate the need for the development of carbon materials that continue to provide sensitive detection while mitigating detrimental fouling.

#### Hormones

Hormones consist of amino acid, peptide, or steroid chemical structures, and, recently, one of the reproductive system’s most notable electroactive hormones, 17*β*-estradiol (E2), has been characterized electrochemically.^96,97^ High concentrations of E2 are produced locally within the brain as a regulator enhancing dopamine receptor expression.^98^ Many analytical techniques to detect E2 are hindered by their slow temporal analysis^99^ resulting in the use of numerous electrochemical detection methods still lacking the temporal capabilities for dynamic E2 detection.^100^ These factors led our lab to explore rapid E2 detection using FSCV to expand our understanding of E2’s function in neurotransmitter regulation.^96^ Our electrochemical characterization of E2 preliminarily supports the need for further improvements as oxygen-modified carbon substrates are not preferential for E2 interactions.^96^

### Limitations & routes of addressing these challenges

Carbon-fiber’s amorphous nature creates difficulty in controlling surface modifications. Tuning the surface of a carbon-based material in terms of surface geometry and chemical functionality/planar nature can hold the key to ideal electrode substrates for neurochemical detection. Poorly defined edge and basal plane sites on CF make uniform modifications with bio-friendly carbon materials nearly impossible as these processes lack the robustness needed for increased success and are reliant on weak electrostatic and pi-pi interactions.^21,43,84^ So, although these carbon nanomaterials possess effective surface and chemical structures, ideal electrode frameworks require full fiber arrangements fabricated from more controllable structures. Three main procedures have recently been developed to implement entirely new surface and geometric structures to replace traditional CFME frameworks opening avenues to tunable carbon materials with the ability to make optimal surfaces for neurochemical interactions (Fig. 8).

The first of these, copolymer wetspinning, consists of a method for surface geometry alterations through pore generation and the ability to create surface chemical hybridization “hot spots” within pore geometries where edge plane sites are localized (Fig. 8A).^25^ These materials were addressed earlier when discussing geometric control; however, we would like to address them here for their utility in improving sensitivity. Small-scale wetspinning is a synthetic method for nanostructure manipulation to CF, but much more optimization to this procedure could lead to more impactful improvements in neurochemical detection. As previously discussed, academic wetspinning has also found utility in other labs with the use of polyethylenimine carbon nanotube fibers for enhanced dopamine adsorption interactions.^38^ The controllability presented by polymer precursor alterations to these fibers prior to carbonization and annealing gives researchers the ability to tune fiber surface properties and grants the ability to dope other carbonaceous materials into the fiber. From the ground up, these wetspinning processes are one of the academic friendly methods we envision used for the future fabrication of surface chemistry/geometry tunable microfibers for enhanced neurochemical detection.

The second approach includes the synthesis of CNT microfiber electrodes for neurotransmitter detection taking advantage of the inherent properties of CNTs like their faster electron transfer capabilities while removing the need for further surface modifications.^35,37,38,84,101^ Perhaps the most important property CNT microfibers possess, especially for FSCV investigations, is their ability to mitigate electrode fouling^35,84,101^ resulting from surface functionality and disorder degree.^35^ The anti-fouling properties of these fibers allow for highly stable measurements of high electroactivity for in-tissue measurements. The mass transport capabilities of CNTs provide low detection limits to various neurotransmitters like dopamine and serotonin^101^ while further improving the temporal resolution of FSCV measurements.^24,27,37^ Unfortunately for CNT fibers, the full synthesis process can be quite strenuous requiring specialized equipment not accessible to all researchers and many of these materials consist of further preactivation/modification. The capabilities of these fibers cannot be ignored, so perhaps the most effective way for them to reach their full potential is to begin to develop new synthetic approaches that are more academically accessible. Commercial availability of these materials has improved recently, ultimately increasing access to those incapable of performing CVD or without an expert collaborator.

The third synthetic method for microfiber tunability is the hydrothermal fabrication of graphene microfibers (Fig. 8B).^44^ Previously discussed, these materials contain various electrical properties that improve their detection as compared to CF while having the ability to tune the surface chemistry for electrochemical measurements. The well-established biplanar chemistry of graphene presents an avenue to understand, experimentally, the surface interactions of neurochemicals at well-defined carbon planes when methods of orienting graphene sheets are used. Graphene is a substrate capable of being doped with various other elements which could allow for enhanced interactions of neurochemicals repelled by ordinary graphene surfaces. Our lab recently developed a method to control the alignment of the edge-plane of GO sheets to promote carefully controlled experiments of neurochemical interactions at the surface.^45^ Preliminary studies show GO sheet alignment plays a vital role in purine detection depleting adenosine interfacial interactions while enhancing that of guanosine at edge plane-aligned GO microfibers.^44,45^ These results support the need for fine-tuned microfibers to investigate and understand electrode-neurochemical interfacial interactions at a molecular level. Unfortunately, graphene microfibers exhibit depleted mechanical properties, so more work is needed for implantation applications and universal neurochemical usage. With that said, we envision the continued optimization and exploration of graphene-based microfibers to present researchers with a means of truly controlled surface chemistry to further advance our understanding of surface interactions, and this has even been supported through atomistic simulations.^102^

The capabilities of simulating interactions present researchers an alternative to traditional experimentation as the computing power of many of these systems permit the user to simulate surface interactions down to single molecules. We believe this will prove extremely useful as the attempt at fabricating sought after tunable carbon substrates requires knowledge into these complex surface interactions to determine the needed tunability for ultra-sensitive surfaces. In a recent study, molecular dynamics was used to simulate the surface interactions of dopamine’s redox couple at the surface of molecularly flat, pristine graphene.^102^ The simulations illustrate, in detail, adsorption interactions at carbon substrates, molecular orientation of the neurochemical at the surface, and can denote the differences in adsorption strength and diffusion rates between the two neurochemicals within the redox couple. Molecular dynamics even show strong adsorption properties of dopamine at the graphene surface without the need of applied potential. This process can lead to simulations of neurochemicals of differing structures and functional groups to model their interactions at pristine graphene or even functionalized carbon surfaces like graphene oxide for an understanding into adsorption of various other neurochemicals of interest. We can also begin to further understand the process of fouling caused by the electropolymerized biproducts of many of the above neurochemicals. In the future, molecular dynamic simulations, with the ability to model individual molecule interactions, will be much needed to couple with fiber fabrication processes to support the controllable surfaces needed for specific neurochemical detection and analyze molecular hinderances in neurochemical/carbon functionality.

## Comparative Remarks

Here, we have provided a review of carbon-based materials to enhance neurochemical detection with FSCV and provided our insight into the directions we envision future innovations taking to continue to expand the capabilities of carbon-based sensing. The novel electrode modifications and new sensing materials discussed in this manuscript are summarized in Table I where we list the notable characteristics of each along with the neurochemical classes they have been used to detect to date with FSCV. CF is unrivaled when it comes to its breadth of uses in FSCV detection of neurochemicals; however, it remains poorly sensitive and selective for many neurochemical classes beyond the catecholamines. A brief review of Table I indicates several neurochemical classes that have been studied, but an overwhelming majority of these innovative materials have been used to further enhance catecholamine detection (notably dopamine). We identify five key characteristics among these novel materials that are improvements compared to CF: improved kinetics and electrocatalysis, fouling mitigation, electrochemical reversibility, improved adsorption, and non-electroactive biosensing.

**Table I.** Summary of carbon-based materials used for the detection of neurochemicals with fast-scan cyclic voltammetry and their notable characteristics.

Carbon-based materials	Notable characteristics	Neurochemical classes detected
CFME	Large working potential window	Catecholamines^1,2,4,5,7^
	Minimally invasive geometry	Purines^47,48,53–55^
	Surface oxide functionality	Indolamines^42,50,85,103^
	Amorphous surface chemistry	Neuropeptides^60,87,89^
	Difficult to tune	Reactive oxygen species^55,91–93^
	Poor selectivity	Metal neurotransmitters^58,59^
		Imidazoles^57^
		Hormones^96^
High Young’s Modulus CF	Improved selectivity	Catecholamines^18^
	Mitigate fouling	Indolamines^18^
	Low sensitivity to anionic metabolites	
CNT-modified CFME	Increased surface roughness	Purines^34^
	Increased sensitivity	Indolamines^85^
	Improved kinetics	Extracellular interferents (ascorbic acid)^34,82^
	Mitigate fouling	
CNT yarn	Mitigates fouling	Catecholamines^24,27,32,33,37,84^
	Enhanced kinetics	Purines^24^
	Improved electrochemical reversibility	Indolamines^24,35,36^
	Frequency independent behavior	Reactive oxygen species^24^
		Extracellular interferents (ascorbic acid)^24^
Porous carbon nanofiber-modified CFME	Improved kinetics	Catecholamines^21^
	Increased sensitivity	
	Increased surface roughness	
Carbon nanospikes	Increased surface roughness	Catecholamines^20,81^
	Increased sensitivity	
	Improved electrochemical reversibility^32^	
	Increased defect sites	
	Increased current density	
	Improved adsorption	
	Metal wire electrodes	
GO-modified CFME	Increased sensitivity	Catecholamines^43^
	Reproducible modification method	
	Improved in vivo measurements	
3D fuzzy graphene modified CFME	High sensitivity	Catecholamines^41^
	Improved selectivity	Indolamines^41^
	Co-detection	
Nanodiamond coated CFME	Improved selectivity	Catecholamines^79^
	Improved limit of detection	Indolamines^79^
	Mitigate fouling	Extracellular interferents (ascorbic acid)^79^
Cavity carbon nanopipette	Frequency independent behavior	Catecholamines^26,27^
	Local trapping	
	Current amplification	
	Increased sensitivity	
GO microfiber	Controllable surface chemistry	Catecholamines^44,45^
	Improved temporal resolution	Purines^44,45^
	Improved electrochemical reversibility	Indolamines^44,45^
	Mitigate fouling	
	Enhanced kinetics	
Porous carbon microfiber	Localized surface chemistry	Catecholamines^25^
	Increased disorder degree	
	Improved electrochemical reversibility	
	Increased linear detection range	
PEDOT/GO coated CFME	Increased sensitivity	Catecholamines^40^
	Improved limit of detection	
	Improved adsorption	
Polyethylenimine/CNT fiber	Improved temporal resolution	Catecholamines^38^
	Improved kinetics	Indolamines^38^
	In-house fabrication	
Glucose oxidase enzyme sensor	Enzymatic production of H_2_O_2_	Reactive oxygen species^61^
	Selective detection	Non-electroactive glucose^61^
	Co-detection	
Lactate oxidase enzyme sensor	Enzymatic production of H_2_O_2_	Reactive oxygen species^62^
	Selective detection	Non-electroactive lactate^62^
	Large probe size	
Electrochemically conditioned CFME	Increase hydroxyl functionality	Catecholamines^5,76,77^
	Increase carbonyl functionality	
	Increased sensitivity	
	Improved kinetics	
Ar-plasma treated CFME	Increased surface roughness	Catecholamines^16^
	Improved adsorption strength	Purines^16^
	Increased sensitivity	
Acid/N_2_-plasma treated CFME	Electrocatalytic properties	
	Improved adsorption	
	Amine surface functionality	
	Improved sensitivity	Purines^15,17^
O_2_-plasma treated CFME	Electrocatalytic properties	Purines^15^
	Improved adsorption	
	Increased oxide functionality	
	Improved sensitivity	
Metal nanoparticle modified CFME	Electrocatalytic properties	Purines^22,23^
	Improved kinetics	
	Increased sensitivity	
	Toxicity	
Nafion-coated CFME	Improved adsorption	Catecholamines^80^
	Increased sensitivity	Indolamines^52^
	Mitigate fouling	
	Electrode surface charge	
Boron-doped diamond microelectrodes		Indolamines^104,105^
		Purines^106^

The five highlighted improvements provided by these carbon-based materials have vastly improved the detection of nearly all neurochemical classes discussed. Numerous materials possess greater conductivity and innate electrical properties than CF providing increased electron-transfer rates (CNTs/Polyethylenimine-CNT fibers, porous carbon nanofibers, porous carbon and GO microfibers, metal nanoparticles); additionally, these materials provide electrocatalytic conversion of multi-step electrochemical redox reactions (i.e., purines—nitrogen acid/plasma doping, oxygen plasma doping, metal nanoparticles). Fouling of CF is detrimental when it comes to tissue implantation, and materials like CNTs, GO microfibers, and Nafion-coated CFMEs mitigate fouling (especially with indolamines) preventing the blockage of electrochemically active sites on the electrode’s surface. The presence of highly defective rich sites (carbon nanospikes, porous carbon nanofiber modifications, porous carbon/GO microfibers) on the electrode’s surface or geometric features capable of local trapping (CNTs, cavity carbon nanopipettes) have illustrated improved electrochemical reversibility of catecholamines. These defect-rich sites and geometric characteristics can even form thin-layer cell type environments generating current amplification and cyclization of catecholamines forming secondary oxidation mechanisms, not observed with cf The emergence of enzyme biosensors has expanded the utility of FSCV detection to some non-electroactive neurochemicals (glucose, lactase, and even glutamate) which are incapable of detection on most carbon-based electrodes (especially CF). Lastly, changes in surface topology (carbon nanospikes, PEDOT/GO coated CFMEs, and Ar-treated, surface roughened CFMEs) and functionality (nitrogen and oxygen doped CFMEs and Nafion-coated CFMEs) have resulted in improved neurochemical adsorption interactions of catecholamines and purines, improving surface coverage and adsorption strength. Although these materials possess excellent characteristics for improving the detection of one or maybe a handful of the neurochemical classes, a universal carbon-based electrode is nonexistent, and we must keep this in mind as we methodically investigate and fabricate these materials in the future. Additionally, the improvements of these novel materials often come with the tradeoff of increased invasiveness, strenuous fabrication methods, toxicity, non-uniformity, etc.

## Outlook

Carbon fiber has remained the gold standard working electrode for real time electrochemical detection of neurochemicals, and rightfully so, due to its biocompatibility permitting rapid biological measurements made over a wide potential window. The advantageous carbon material has paved the way for extensive dynamic monitoring of dopamine vastly improving our understanding of the signaling mechanism and its role in regulating natural biological processes, but this has led to poor interfacial interactions of many other important neurochemicals. This oversight can be attributed to carbon fiber’s amorphous chemical structure resulting in insufficient molecular affinity to other neurochemical structures creating measurements impacted by mechanistic side products and poor sensitivity. This critical review of carbon-based neurochemical detection has provided an in-depth analysis of the limitations of these pioneering materials. Namely the need for improved dynamic detection, the poor surface chemistry of CF hindering molecular specificity, and lacking robustness in surface modifications. The inability to address these challenges through the carbon-based materials themselves has led to novel work in surface treatments and voltammetric waveform alterations to overcome these challenges. We believe many of these solutions can be further improved with individualized carbon materials specific to neurochemicals of interest.

Materials of increased electrical conductivity and enhanced surface geometry will improve the speed of these dynamic measurements providing fully temporally resolved measurements. A major gap caused by the novel materials of the past is the need for more accessible synthetic procedures to overcome the inaccessibility to advanced laboratory equipment needed for these advanced carbon structures. In depth studies on the mechanisms of neurochemicals other than dopamine have pointed out the need to address the poor surface chemistry of CF for improved selectivity using improved electrocatalytic and elementally doped materials coupled with computational modeling. These two routes together can provide insight to single-molecule interactions at carbon-based materials and provide a roadmap to required surface chemical structures for improving detection of understudied, lowly sensitive neurochemicals. Heterogeneity of carbon fiber’s surface chemistry makes uniform modification tunability challenging as exposing specific plane interactions is impossible. In recent years we have witnessed the emergence of diamond^104–110^ and CNT hybrid^24,27,33–35,38,82,84,88,101^ materials for improved electrochemical performance. These innovative materials have also found utility outside of the FSCV community, and researchers have fabricated hybrid electrode materials used to investigate neurochemicals with more traditional electrochemical detection techniques.^111,112^ We believe the well-defined chemical structures of materials like diamond and CNTs exhibited in these hybrid structures could pose interesting utility to exploring neurochemicals with low sensitivity with FSCV. Additionally, by improving throughput in graphene oxide sheet alignment and further investigation of wetspun carbon fiber frameworks one would have the ability to improve detection temporal resolution, redox cycling, and fiber mechanical properties with highly localized single chemical plane areas. The ability to couple these novel fibers with the support of molecular dynamic simulations will enable the fabrication of highly sensitive carbon-based materials that are tunable and easily controlled. Overall, we envision these avenues as key future directions of addressing CF limitations to push the boundaries of overall neurochemical detection and branching out from dopamine analysis.

In conclusion, this critical review provides directions to improve overall neurochemical detection electrochemically with carbon-based materials. The insight given provides a means for advancing carbon substrate’s synthetic feasibility, dimensional compatibility, and surface chemical connectivity to expand our understanding of dynamic signaling of an array of neurochemicals that have been vastly understudied. Ultimately, we believe if the insights of this critical review are addressed, neurochemical researchers could produce synthetic routes allowing for individualized fabrication of electrodes correlated to specific neurochemicals of interest. Recent novel material innovations have proven helpful in improving the three main limitations mentioned, but we believe a predominant focus on moving away from amorphous carbon fiber could hold the key to advancing the current state of the neurochemical detection field. This would allow us to broaden our understanding of overall real-time neurochemical signaling and improve our overall understanding of neuro-communication using materials capable of capturing neurochemical real-time release during homeostasis or detrimental neurological events.
